# The Genetics of Host Plant Acceptance in Pea Aphids

**DOI:** 10.1111/mec.17795

**Published:** 2025-05-15

**Authors:** Isobel Eyres, Hannah Fenton, Jean Christophe Simon, Jean Peccoud, Julia Ferrari, Roger Butlin, Carole M. Smadja

**Affiliations:** ^1^ School of Biosciences The University of Sheffield Sheffield UK; ^2^ Fera Science Ltd York UK; ^3^ INRAE Rennes France; ^4^ Universite de Poitiers UFR Sciences Fondamentales et Appliquees, EBI Poitiers France; ^5^ Department of Biology University of York York UK; ^6^ University of Montpellier, Institut Des Sciences de L'evolution de Montpellier Montpellier France

**Keywords:** Genomics/Proteomics, Invertebrates, Population Genetics – Empirical, Speciation

## Abstract

The evolution of host‐associated sympatric populations in phytophagous insects (so called “host races”) connects adaptive divergence to barriers to gene flow. Pea aphid ( *Acyrthosiphon pisum* ) host races specialise on legume species, and because host plant choice leads to assortative mating, the genetic basis of host plant acceptance is key to understanding speciation. Aphids use smell and taste in their host plant selection. While chemosensory genes frequently emerge as “outliers” in genome scans, their link to plant acceptance behaviour remains unclear. We examined the genetic basis of host‐associated phenotypes using an F2 cross between two pea aphid host‐associated races (specialised on alfalfa— *Medicago sativa* ‐ and pea— *Pisum sativum* ), assaying behaviour on both host plants and conducting QTL and regional heritability analyses based on a high‐resolution linkage map. We identified five regions of moderate effect associated with acceptance of alfalfa, two with pea acceptance and two with survival on alfalfa. Two QTLs, one for alfalfa and one for pea acceptance, are located within a large rearranged region on chromosome 1, while other QTLs linked to alfalfa acceptance and survival are in the same region on chromosome 3—linking host plant choice to fitness. These findings highlight the polygenic basis of acceptance behaviour and the role of gene clustering and chromosomal rearrangements in promoting coupling among barrier loci. We identified 60 chemosensory genes within regions connected to acceptance, 24 of which were divergent among pea aphid races in previous genome scan or gene expression analyses. Evidence linking these genes to acceptance phenotypes supports their role in determining host plant specificity and as barrier loci contributing to pea aphid speciation.

## 1 Introduction

Speciation is the process by which genetically connected populations diverge into reproductively isolated species (Butlin 2024), and this process depends on the evolution of barriers to gene flow. It is now widely accepted that gene flow between diverging populations is common during the speciation process (Abbott et al. 2013), so understanding how speciation can progress despite ongoing gene flow is an important challenge for speciation research. Natural selection can be an important driver in this process, as local adaptation can initiate reproductive isolation in the face of gene flow, resisting the influx of non‐adaptive alleles. However, in cases where reproductive isolation becomes strong, further isolating barriers, and in particular those generating assortative mating, must evolve (Kulmuni et al. 2020; Rundle and Nosil 2005). As such, a key step in our understanding of speciation is to determine how local adaptation and assortative mating can become coupled despite the presence of gene flow (Butlin and Smadja 2018; Smadja and Butlin 2011).

Ecological speciation is particularly likely where exploited habitats and reproduction are inherently connected, as in the case of phytophagous insects which mate on their chosen host plants (Bush 1969). Mating on host plants generates assortative mating among insects with the same host‐plant preference. Understanding the genetic and genomic basis of host plant choice and how the genetic architecture of this phenotype promotes its co‐evolution with performance phenotypes on host plants directly under selection (via pleiotropy or indirect selection promoted by physical linkage) is therefore an important step in understanding how barriers to gene flow arise and spread in the genome during the process of speciation (Drès and Mallet 2002; Via 2001).

One such example of speciation in phytophagous insects is the pea aphid ( *Acyrthosiphon pisum* ). This aphid species comprises at least 15 genetically distinct host races or ‘biotypes’ in Europe, existing in sympatry in some areas of their distribution ranges and specialising on different legume species (Peccoud et al. 2015; Simon and Peccoud 2010). Host plant specialisation was previously evidenced in pea aphids from the US and Europe by their differential performance (survival, fecundity) on home versus non‐home plants, their preferential behavioural acceptance of their home plant for feeding and reproduction and their preference for their home plant in choice experiments (Caillaud and Via 2000; Ferrari et al. 2006, 2008; Peccoud et al. 2009a; Via and Hawthorne 2002). Estimates of the age of the pea aphid radiation vary widely, from 18,000 to 47,000 years when based on the divergence of their obligate endosymbionts *Buchnera aphidicola* (Peccoud et al. 2009b) to 419,000–772,000 years when based on nuclear divergence (Fazalova and Nevado 2020), and there is currently gene flow among most host races (Peccoud et al. 2009b), suggesting that the pea aphid complex is an ongoing adaptive radiation.

Because aphids mate on their preferred host plant, aphid selection of host plants has the potential to form an important pre‐mating isolating barrier. We know that pea aphid host plant choice is a multi‐step process (Powell et al. 2006), involving both smell and taste (Nottingham and Hardie 1993). Peripheral recognition of odour and taste molecules involves a large set of chemosensory genes (CSGs) in aphids, including gustatory receptors (GRs), odorant receptors (ORs), ionotropic receptors (IRs), odorant binding proteins (OBPs), chemosensory proteins (CSPs) and sensory neuron membrane proteins (SNMPs) (Eyres et al. 2017; Robertson et al. 2019; Smadja et al. 2009). As of today, there is currently limited functional evidence for the role of these genes in aphids (but see Zhang et al. 2017 for an example).

Multiple studies of the genetics of divergence between pea aphid host races point to the involvement of chemosensory genes: using a candidate gene scanning approach (Smadja et al. 2012) identified 18 chemosensory outlier genes (5 GRs, 13 ORs) when comparing *Lotus*, *Medicago* and *Trifolium* host races, while a whole genome scan approach by Nouhaud et al. (2018) identified 25 chemosensory genes (14 GRs, 10 ORs and 1 CSP) associated with genomic hotspots of divergence between *Medicago, Trifolium* and *Pisum* races, and targeted resequencing (Eyres et al. 2017) confirmed the divergence of large numbers of CSGs across multiple host races and geographic scales. Furthermore, Eyres et al. (2016) identified 32 chemosensory genes differentially expressed by race or by host plant when comparing six host races (4 CSPs, 6 GRs, 3 IRs, 4 OBPs, 11 ORs and 4 SNMPs). These studies provide a set of potentially interesting and important chemosensory genes, confirming the value of further investigation of this gene category in aphids. However, this type of broad genomic study is prone to generate false positives and does not provide a direct link between genotype and phenotype (Ravinet et al. 2017; Wolf and Ellegren 2017).

Identifying the genes underlying specific phenotypes involved in reproductive isolation not only provides us with information about the evolutionary mechanisms that promote divergence, it can also reveal the underlying genetic architecture of barrier traits. This facilitates the testing of hypotheses about the accumulation of different types of barriers and the generation of linkage disequilibrium among underlying loci. Identifying barrier genes in pea aphids representing a continuum of divergence (Peccoud et al. 2009b) provides a particularly good system for investigating the build‐up of barriers to gene flow. Given the uncertainties associated with outlier detection (e.g., Hermisson 2009), it is important to integrate quantitative genetics approaches to more directly link genotype and phenotype and to confirm outliers by alternative means (Stinchcombe and Hoekstra 2008).

To this aim, we decided to use a QTL mapping approach in the pea aphid to connect host acceptance behaviour to specific regions of the genome and test for an association between acceptance and performance phenotypes at the genetic level. Here, we examined the genetic basis of host plant acceptance and survival on host plants by generating an F2 cross between two *A. pisum* host races respectively specialised on *Medicago sativa* and *Pisum sativum* , assaying behaviour on both host plants, RAD sequencing the offspring and conducting QTL and regional heritability analyses to link regions of the genome to plant acceptance behaviour and survival phenotypes. We generated a high‐resolution linkage map to increase the number of markers available and to connect them more directly to the available genome assembly, compared to previously published linkage maps (Hawthorne and Via (2001), 173 markers; Jaquiéry et al. (2014), 305 markers). By placing chemosensory genes onto our high‐density linkage map, our objectives were to test whether specific chemosensory genes relate to acceptance QTLs identified and whether previously identified outlier genes map within regions associated with plant‐acceptance behaviour. This approach enabled us to establish the genetic architecture of barrier traits: the number of loci underlying a trait, the distribution of QTLs across the genome and to assess the role of genomic rearrangements in the divergence of pea aphid host races. By looking for QTLs relating to survival on the different host plants, we also aimed to test how the different barriers to gene flow of acceptance behaviour and survival are connected in the genome and to ask whether categories of genes potentially involved in performance on host plants (detoxification genes or salivary effectors (Boulain et al. 2019; Boulain et al. 2018; Lu et al. 2016; Simon et al. 2015; Vertacnik and Linnen 2017)) lie close to genes expected to be associated with host plant acceptance (chemosensory genes).

## 2 Materials and Methods

### 2.1 Experimental Cross Design

Pea aphids ( *Acyrthosiphon pisum* Harris) are facultative sexuals and will reproduce asexually as long as they are maintained under long‐day light conditions, enabling the growth and maintenance of clonal populations in the laboratory. Prior to the experiments, the aphids were kept on *Vicia faba* (var. Sutton Dwarf), a host plant on which almost all pea aphids perform well (Ferrari et al. 2008; Peccoud et al. 2014). The aphids were reared at 15°C and 60 ± 15% relative humidity and a 16 h light: 8 h dark cycle unless described otherwise below.

**FIGURE 1:**
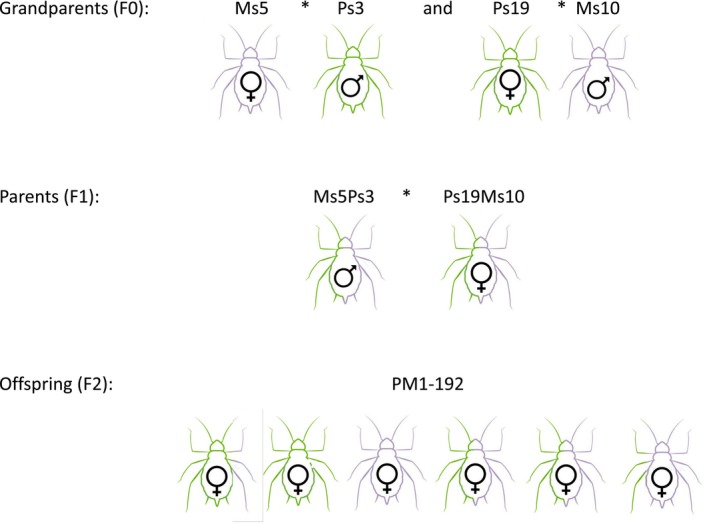
F2 mapping cross. Ms5 = *Medicago sativa* biotype clone 5, Ms10 = *Medicago sativa* biotype clone 10, Ps3 = *Pisum sativum* biotype clone 3, and Ps19 = *Pisum sativum* biotype clone 19.

An F2 mapping cross was established (Figure 1). In the F0 generation, a *Medicago* biotype female (Ms5—sampled near Lantenay, eastern France (46°03′ N, 5°32′ E)) was mated with a *Pisum* biotype male (Ps3—sampled in Le Rheu, northwestern France (48°06′ N, 1°47′ W)) and a *Pisum* biotype female (Ps19—sampled in Saint‐Prex, Switzerland (46°29′03.3″ N, 6°26′40.9″ E)) was mated with a *Medicago* biotype male (Ms10—sampled near Lantenay, eastern France (46°03′ N, 5°32′ E)). To generate the F2 generation, male morphs were induced for the Ms5Ps3 clone from the first cross and female morphs from the Ps19Ms10 clone from the second cross. This was done by rearing sexuparae (the parents of sexual morphs) at 18°C and gradually reducing day length over a period of 5 days, from 14 to 13 h for the production of males and from 12 h 30 min to 12 h 12 min for females. The aphids treated in this way gave birth to the sexual morphs (Via 1992). Once these were adults, 54 Petri dishes each with 2 males from the Ms5Ps3 clone and 3 females from the Ps19Ms10 clone on a leaf of *V. faba* were set up to produce the F2 eggs. Eggs were collected over a period of eight days, surface sterilised with 1% bleach and kept in Petri dishes on moist filter paper at 4°C and a 16 h light: 8 h dark regime for 2 months until they started to hatch. This resulted in 192 F2 clones (o1‐o192) that were genotyped and characterised for their acceptance of and survival on *Medicago sativa* and *Pisum sativum*.

### 2.2 Phenotyping

All 192 F2 offspring, plus the two F1 parents and four F0 grandparents, were assayed for behaviour on alfalfa ( *M. sativa* ) and pea ( *P. sativum* var. Hurst Greenshaft) plants. Because aphids can reproduce by clonal reproduction, it was possible to test multiple replicates of the same genotype. Four replicates per genotype were assayed on each plant species. The behaviour assay was performed on a different day for each of the four replicates per genotype. For each replicate, one seven‐day‐old fourth‐instar aphid grown on *V. faba* was placed onto a leaf of the test plant that was stuck upside down into a Petri dish filled with agar to ensure that only the abaxial surface (the side that pea aphids are usually found on) of the leaf was accessible and the aphid could be monitored. The aphid was observed after 1, 2, 3, 4, 5, 10, 15, 20, 25 and 30 min and 1, 2, 3, 4, 23, 24 and 25 h. At each observation, it was recorded whether an aphid was probing (i.e., inserting the stylet), not probing (i.e., not inserting the stylet) or dead.

Pea aphid behaviour on each plant species (pea and alfalfa) was summarised into four phenotypes: acceptance of pea, acceptance of alfalfa, survival on pea and survival on alfalfa. For the acceptance phenotype, we took probing as a proxy of ‘acceptance’ (Caillaud and Via 2000; Powell et al. 2006) and divided this by observations where the aphid was alive and therefore really able to accept or reject. For the survival phenotype used in our r/QTL analysis, we calculated mean time steps alive. These phenotype measures were averaged over all time points of the four replicates. We used an alternative measure of survival in our regional heritability analysis: count of aphids alive at the end of the experiment, out of the four trials for each F2 clone.

### 2.3 DNA Extraction, Library Prep and Sequencing

With the aim of using RAD sequencing to genotype F2 aphids and establish a linkage map, DNA was extracted from the 192 F2 clones, their parents (Ms5Ps3 and Ps19Ms10) and grandparents (Ms5, Ps3, Ms10 and Ps19). DNA was extracted from whole clonal female aphids using Machery Nagel Nucleospin DNA extraction kit (standard protocol) and 5–30 μg DNA per sample was sent for library preparation and sequencing by Edinburgh Genomics. Single digest RAD library preparation was performed as in Baird et al. (2008), using *Pst*1. DNA samples were barcoded, and then 24–25 clones were pooled per sequencing lane and sequenced using Illumina HiSeq v4 paired‐end sequencing (125 bp read length, 300 bp target insert size).

### 2.4 Processing Sequencing Data—Quality Filtering and Mapping

Sequencing adapters were removed by Edinburgh Genomics. Library quality was checked using FASTQC (http://www.bioinformatics.babraham.ac.uk/projects/fastqc). The Stacks process_radtags module was used to demultiplex pooled reads, remove barcodes, remove any sequence with an uncalled base and to discard reads with low quality scores (base call accuracy < 90% (phred < 10) for a window size of 15% of read length). The stacks clone_filter module was used to remove PCR duplicates. Paired‐end reads were then mapped to a chromosome‐level pea aphid genome assembly (pea_aphid_22Mar2018_4r6ur, PRJNA496478; Li et al. 2019) using BWA‐MEM with default parameters. Four F2 individuals (o92, o172, o135, o154) were excluded from further analyses due to very low read counts (< 0.5 million).

### 2.5 Linkage Mapping With Lep‐MAP3

Linkage maps were constructed using Lep‐MAP3 (Rastas 2017). Genotype calling and conversion of markers to LepMap3 input format was performed using the standard LepMap3 input pipeline composed of mpileup (samtools), PileupParser2.awk (LepMap script) and Pileup2posterior.awk (LepMap script). We used default parameters except for minimum mapping quality of 30 and minimum base quality of 10 in mpileup and depth of coverage per individual between 5 and 50 in PileupParser2. Markers were further filtered using a custom r script (archived on zenodo, Eyres et al. (2025)) to include only markers with homozygous host race specific genotypes in F0s and heterozygous genotypes in F1s before inputting into the LepMap3 main pipeline.

To call missing/erroneous parental genotypes, ParentCall2 was used on the complete dataset of grandparents, parents and offspring. Filtering2 was used to remove markers with significant segregation distortion (data Tolerance = 0.001). Linkage groups were identified using the SeparateChromosomes2 module, which was initially run with lodLimits from 1 to 50 to identify the most appropriate LOD threshold. *A. pisum* has four chromosome pairs (three autosomes and an X chromosome). The expected four linkage groups were produced with lodLimits 31–43. SeparateChromosomes2 was then run with lodLimit = 31, distortionLod = 1 and sizeLimit = 20 (excluding linkage groups containing < 20 markers). JoinSingles2All was run with lodLimit = 3 to assign singular markers to existing LGs, and markers with incongruencies between linkage group assignment and genomic chromosome location, as well as markers incorrectly showing recombination in males (there is no crossing over in *A. pisum* males) were removed. OrderMarkers2 was then run to order markers within each linkage group separately; we ran OrderMarkers2 10 times, and markers showing inconsistent map positions were removed. The final reduced subset of 6443 markers was used to generate the final linkage map.

To identify genomic rearrangements, the genetic position of each SNP in the female linkage map was plotted against its physical position in the Li et al. (2019) (v3) genome assembly to generate Marey maps of each of the four linkage groups. Rearrangements were identified as regions deviating from the monotonically increasing trend expected from a Marey map, and as regions with evidence of incongruous locations between the genomic and linkage maps. Regions of suppressed recombination were identified as runs of markers spanning a range of genomic positions but showing no change in linkage map position.

### 2.6 QTL Mapping

QTL analysis was performed using the R/qtl package (Broman et al. 2003). The set of 6443 markers for the linkage map was further screened and filtered, firstly using a custom r script to remove redundant markers (i.e., retaining a single marker per linkage map position) and then using the best‐practice guidelines outlined in the R/qtl tutorial (https://rqtl.org/tutorials/geneticmaps.pdf). After using recombination fraction plots to identify ambiguously placed markers and removing markers with error lod > 5, 484 markers remained for the final QTL analysis: 170 markers on chromosome A1, 112 on chromosome A2, 62 on chromosome A3 and 140 on the X chromosome (chromosome names designated based on the Li et al. (2019) genome assembly).

For all phenotypes, we performed standard interval mapping using the filtered set of 484 markers with a single QTL model. As the acceptance phenotype was not normally distributed, a non‐parametric approach was applied. The survival phenotype showed a spike in the distribution for individuals who survived to the end of observations. To take this into account, we applied a two‐part model as described in Broman and Speed (2002) (model = “2part”, upper = TRUE). After calculating genotype probabilities, the *scanone* function was used to estimate QTL LOD scores. Permutation tests with 1000 permutations were used to test the significance of QTLs and to estimate confidence limits on QTL positions. The 95% confidence interval of a QTL detected was estimated by a 1.5‐LOD drop method using the *lodint* function. The percentage of phenotypic variance explained by QTLs was calculated using the equation: 1–10^−(2/*n*)×LOD^.

### 2.7 Regional Heritability Analysis

Regional heritability mapping was introduced as a complement to single‐marker analyses on genome‐wide association studies (Nagamine et al. 2012). It can improve the detection of genome regions influencing quantitative traits (Caballero et al. 2015) and is now widely used (e.g., Hillestad et al. 2020; Koch et al. 2021; Peters et al. 2022). The approach tests whether adding a genetic relatedness matrix based on a genomic window accounts for additional variance in a trait compared to a model using only the genome‐wide relatedness matrix, and by doing, so it can enable the identification of regions that cannot be detected by standard methods (James et al. 2024). It can also be applied to an F2 family, although we are not aware of any such analysis, and may detect genomic regions that contribute to variation in a trait but are missed by single‐QTL analyses, for example the region contains multiple QTL, each of which has too small an effect to be detected alone. Since chemosensory genes are known to be clustered in the pea aphid genome (Smadja et al. 2009), such clustering of small‐effect QTL is possible for host acceptance.

For this analysis, we used the brms package in R (Bürkner 2017) to fit two alternative models. The first included only the genome‐wide relatedness matrix for the F2 individuals, based on all SNPs used in the QTL mapping. This provided an estimate of the overall heritability. The second included two random terms, one for a relatedness matrix based on SNPs in a 20 cM genomic window and one for all other SNPs. From the second model, we extracted an estimate of the regional component of the additive genetic variance, *Vr*. Relatedness matrices were obtained with the AGHmatrix package (Amadeu et al. 2023). The analysis was repeated for 20 cM sliding windows across the genome (number of SNPs per window: mean = 16, 80% of windows with > 10 SNPs), with a 10 cM slide. It was applied to acceptance on either pea or alfalfa, as described above, and to survival on pea or alfalfa. For acceptance, we used a Gaussian error distribution. For survival, we could not use the two‐part model applied in R/qtl. Instead, we used the count of aphids alive at the end of the experiment, out of the four trials for each F2 clone, and applied a binomial error distribution.

We applied a permutation test for each genomic window, following Nagamine et al. (2012). This test retained the relationship between individuals and traits and between individuals and the genomic background, but permuted the relationship between individuals and the regional relatedness matrix. In this way, the permutations retained the true heritability of the trait, but no regional contribution was expected. We ran 1000 permutations for each trait and genomic window and used the 95th percentile as a suggestive cut‐off. Since we tested 57 overlapping genomic windows, this is a lenient criterion that is likely to result in some false positives.

### 2.8 Placement of Chemosensory Genes on Genomic and Linkage Map and Association With QTLs

**FIGURE 2:**
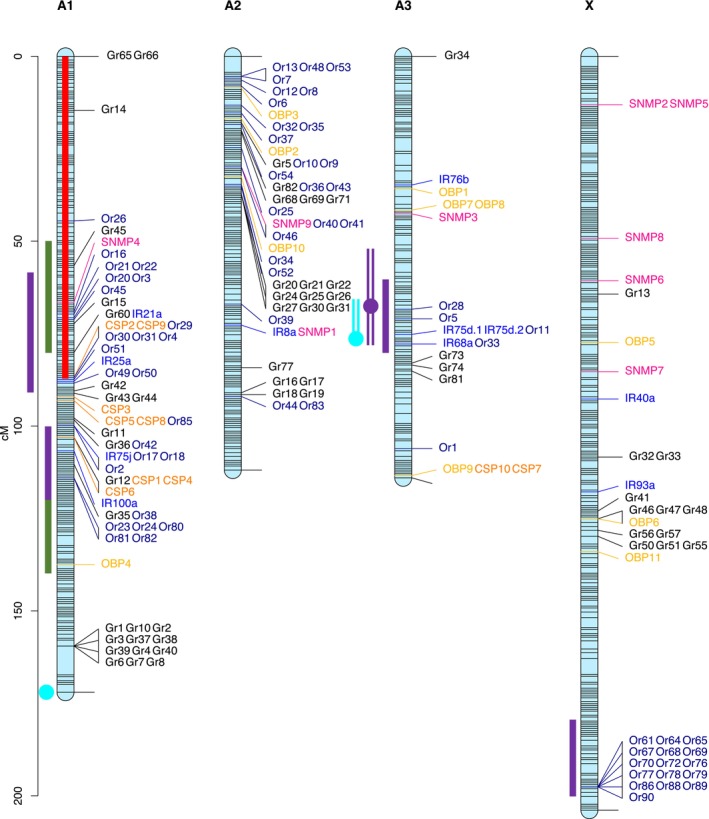
Pea aphid linkage map. Locations of chemosensory genes shown: Or (dark blue) = olfactory receptors, Gr (black) = gustatory receptors, OBP (dark yellow) = olfactory binding proteins, CSPs (orange) = chemosensory proteins, IRs (blue) = ionotropic receptors and SNMPs (pink) = sensory neuron membrane proteins. Red bar shows location of putative inversion relative to the LSR1 pea aphid strain. Dark green bars show location of acceptance of pea regional heritability blocks, dark purple bars show location of acceptance of alfalfa regional heritability blocks, dark purple open bars show location of acceptance of alfalfa QTL (dot marks centre of the qtl), cyan open bars show location of survival on alfalfa QTL (dot marks centre of the qtl).

Chemosensory genes from the following categories were identified in the pea aphid genome (Li et al. 2019), assembled to the chromosomal level: gustatory receptors (GRs), olfactory receptors (ORs), ionotropic receptors (IRs), chemosensory proteins (CSPs), odorant binding proteins (OBPs) and sensory neuron membrane proteins (SNMPs). Updated gene models from Robertson et al. (2019) were used for GRs, ORs and IRs, while sequences for OBPs, CSPs and SNMPs were those identified in Smadja et al. (2012). Each sequence was searched for using blastn (Altschul et al. 1990) against the Li et al. (2019) *A. pisum* assembly on genbank (GCA_005508785.2). In total, 179 annotated chemosensory genes (70 ORs, 60 GRs, 19 IRs, 11 OBPs, 10 CSPs and 9 SNMPs) were placed on the four main chromosomal scaffolds (Figure 2). Each chemosensory gene could then be mapped to its closest marker on the linkage map, and the chemosensory genes lying within each QTL or significant regional heritability block could be identified. We used the Bioconductor *RegioneR* package (Gel et al. 2016) in R to test using permutations for an over‐representation of chemosensory genes within significant QTL or regional heritability blocks associated with acceptance phenotypes (outlier CSGs resampled 10,000 times among all chemosensory annotated genes). Fisher‐exact tests were used to assess the enrichment of some categories of chemosensory genes among all chemosensory genes lying within QTL/regional heritability regions.

## 3 Results

### 3.1 Sequencing

We obtained RAD sequencing data for four grandparents (Ms5, Ps3, Ps19 and Ms10), two parents (Ms5Ps4 and Ps19Ms10) and 192 F2 offspring (o1‐o192) in our F2 mapping cross (Table S1). The average total number of reads was 3.4 million, and the average properly paired reads mapping to the Li et al. (2019) genome was 3.02 million. Ms5Ps3 (one of the two F1s) had a relatively low total mapped (574,054), but we retained this sample because of its important position in the pedigree. LepMap3 was used to reconstruct missing parental genotypes using the ParentCall function.

### 3.2 Linkage Map

A high density linkage map was generated, comprising four chromosomes, which we named in line with Li et al. (2019): A1 (contig 20849/NC_042494.1/CM016664.1), A2 (contig 21,967/NC_042495.1/CM016666.1), A3 (contig 21646/NC_042496.1/CM016667.1) and X (contig 21773/NC_042493.1/CM016665.1) (Table S2). The total map length was 602 cM (A1 = 172 cM, A2 = 112 cM, A3 = 114 cM and X = 204 cM). The 6443 markers placed on this linkage map were distributed across 790 map positions as follows: 2181 markers at 234 unique positions on A1, 2213 markers at 168 unique positions on A2, 592 markers at 129 unique positions on A3 and 1457 markers at 259 unique positions on X (archived on zenodo, Eyres et al. (2025)) (Figure 2).

**FIGURE 3:**
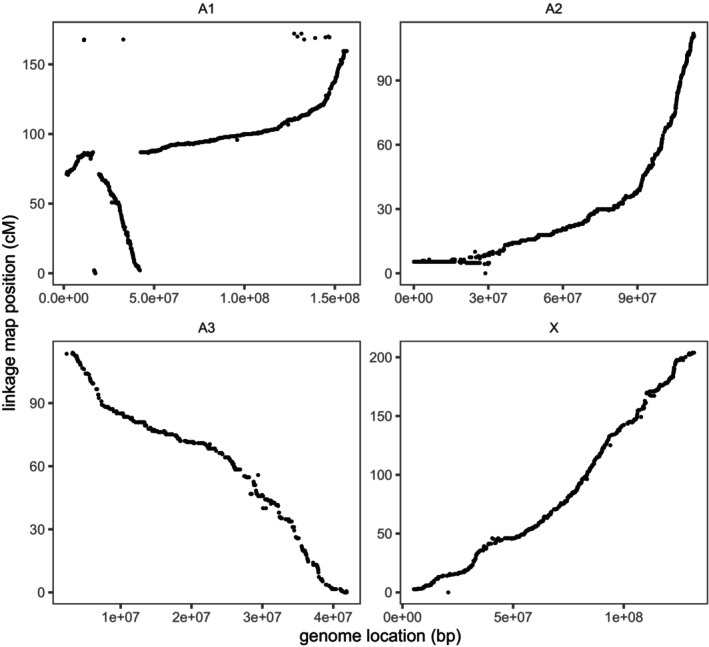
Marey maps plotting the genetic position of each SNP in the linkage map against its physical location on the Li et al. 2019 (v3) genome assembly.

**FIGURE 4:**
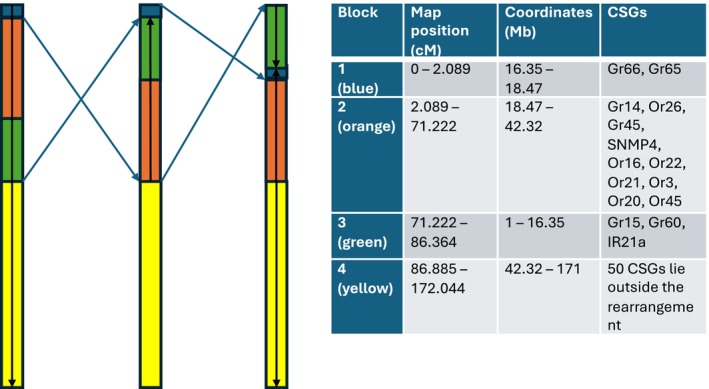
Diagram of the rearranged region on chromosome A1.

Comparing the linkage map to physical positions on the Li et al. (2019) genome (Figure 3) and to the linkage map published in Jaquiéry et al. (2014) shows that chromosome A1 contains a large rearrangement. This 86 cM rearrangement can be split into three blocks: block 1 (0–2 cM on the linkage map), block 2 (2–71 cM on the linkage map) and block 3 (71–86 cM on the linkage map) can be found in the order: block 3 (same direction), block 1 (inverted), block 2 (inverted) in the genome. This requires a minimum of two inversion events (Figure 4). We infer that this rearrangement must have been homozygous in the female F1 parent, and so present in both host races, resulting in no recombination suppression in the generation of the F2 family. We cannot say whether the inversion is segregating or fixed within the pea race, but it does appear to be segregating within the alfalfa race, which also includes the genome reference clone. There is also evidence of a large region of reduced recombination at the start of chromosome A2, most likely due to an inversion that was heterozygous in the F1 female (Figure 3). This rearrangement could be fixed differently between the host races or segregating within one or both of them.

### 3.3 Acceptance and Survival Phenotypes

F0 aphids from the two host races showed a preference for their home plant on the basis of the acceptance phenotype. On alfalfa plants, aphids of the alfalfa race showed a significantly higher mean acceptance score (0.463) than aphids of the pea race (0.213) (Mann–Whitney U, W = 52, *p* = 0.040), while F1 and F2 aphids both showed intermediate mean acceptances of 0.345 and 0.409, respectively. On pea plants, F0 aphids of the pea race showed a higher mean acceptance score (0.602) than aphids of the alfalfa race (0.500) but this difference was not significant (Mann–Whitney U, W = 26, *p* = 0.5624), while F1 and F2 aphids both showed elevated mean acceptances of 0.802 and 0.677, respectively (Figure S1).

There was no difference in survival of the two F0 clones when trialled on pea plants (average survival = 16.6 time steps for both alfalfa and pea F0s), but a marginally greater average survival on alfalfa for the alfalfa‐derived aphids (average 17 time steps) in comparison to the pea‐derived aphids (average 16.6 time steps). F1 and F2 aphids on alfalfa showed a slightly reduced mean survival (15.9 and 16.9 time steps respectively), while on pea F1 and F2 aphids showed a very similar survival to the F0s (16.3 and 17.0 time steps, respectively) (Figure S1).

### 3.4 QTL Mapping and Regional Heritability Analysis

Standard interval mapping was performed in r/QTL using the set of 484 stringently filtered markers (170 on A1, 112 markers on A2, 62 on A3 and 140 on the X) for each of the following variables: acceptance of alfalfa, acceptance of pea, survival on alfalfa and survival on pea. Using r/QTL, three significant QTLs were identified (Table S3; Figure S2). These comprise one significant QTL for acceptance of alfalfa (Table 1) (chromosome A3, position 68, explaining ~15% of phenotypic variation) and two for survival on alfalfa (chromosome A1, position 171, explaining ~10% of phenotypic variation; chromosome A3, position 77, explaining ~7% of phenotypic variation). Notably, the single QTL for acceptance of alfalfa and one of the two QTLs for survival on alfalfa mapped to overlapping regions of chromosome A3 (Figure 2). No significant QTL was identified for either acceptance or survival on pea plants.

**TABLE 1:** Genomic and linkage map coordinates of QTL/RHB regions associated with acceptance of host plant, and chemosensory genes located within these regions.

QTL/RHB regions identified as associated with acceptance of host plant	Chr	Coordinates (cM)	Coordinates (Mb)	Within rearranged region on A1?	Total number of CSGs	Number of GR	Number of OR	Number of IR	Number of OBP	Number of CSP	Number of SNMP	Name of CSG outliers consistently found in this study and previous studies*
RH acceptance of pea	A1	50–80 cM	1–7.62 (71–80 cM); 18.47–30.19 (50–71 cM)	Yes	**11**	3	6	1	0	0	1	**Gr45**, SNMP4, Or16, Or22, **Or21**, **Or3, Or20**, Or45, Gr15, Gr60, IR21a
RH acceptance of alfalfa	A1	60–90 cM	1–16.35 (71–86 cM); 18.47–25.16 (60–71 cM); 42.32—56.0 (86–90 cM)	Yes	**20**	2	13	2	0	2	1	SNMP4, Or16, Or22, **Or21**, **Or3, Or20**, Or45, Gr15, Gr60, IR21a, Or30, CSP2, CSP9, Or29, Or51
RH acceptance of alfalfa	A1	100–120 cM	104.54–141.94		**13**	2	6	2	0	3	0	Or38, Gr35, Or24
RH acceptance of pea	A1	120–140 cM	141.94–150.55		**1**	0	0	0	1	0	0	OBP4
r/QTL acceptance of alfalfa	A3	52–78 cM	13.92–29.4		**9**	0	4	5	0	0	0	Or28, Ir75d1, Ir75d2
RH acceptance of alfalfa		60–80 cM	12.11–25.96									
RH acceptance of alfalfa	X	180–200 cM	120.32–127.9		**16**	0	16	0	0	0	0	Or65

* Contains any CSGs ID'd in at least one of the comparison studies. Green font for pea phenotypes, Purple for alfalfa phenotypes. **Bold** for those ID'd in 3 or 4 others.

Mean heritability was calculated for each phenotype, estimated as the proportion of phenotypic variation explained by the genome‐wide relatedness matrix: mean heritability for acceptance of pea was 0.097 (95% plausible interval 0.0002–0.39), mean acceptance of alfalfa was 0.299 (0.094–0.576), mean survival on pea was 0.388 (0.0019–0.828) and mean survival on alfalfa was 0.148 (0.00015–0.574). We calculated the statistical power to detect a QTL, given these heritabilities, following the methods of Hu and Xu 2008), on the basis of a population size of 192 F2s, an average marker distance of 0.8 cM, and a type I error rate alpha of 0.01. The power to detect a single QTL for acceptance of pea with a heritability of 0.097 is 0.97, the power to detect a QTL for acceptance of alfalfa with a heritability of 0.299 is 1, the power to detect a QTL for survival on pea with a heritability of 0.388 is 1, and the power to detect a QTL for survival on alfalfa with a heritability of 0.148 is 1. If any of these phenotypes were underpinned by a single QTL responsible for explaining all of the heritable variation, then we would have expected, with high confidence, to be able to identify them. However, if instead each behaviour were underpinned by just 10 QTLs of equal effect, for example, the power to detect these QTLs would be much lower (acceptance of pea = 0.110, acceptance of alfalfa = 0.423, survival on pea = 0.559 and survival on alfalfa = 0.181).

We did detect QTLs of moderate effect explaining ~15% of variance for acceptance of alfalfa and 10% and 7% of variance for survival on alfalfa. The absence of QTLs for acceptance or survival on pea, despite quite high power to detect a single QTL for a locus of their heritabilities, suggests that the heritable genetic variation in behaviour or survival on pea comes from at least a few loci of small effect, rather than a single locus.

Our regional heritability analysis (Nagamine et al. 2012), performed in order to increase our power to detect loci of smaller effect, suggested four regions associated with acceptance of alfalfa (Table 1). These include two regions mapping to chromosome A1 (60–90 cM and 100–120 cM), one mapping to the same location as the QTL identified using r/qtl (60–80 cM on chromosome A3), and another at the end of the X chromosome (180–200 cM). In contrast to r/qtl, which identified no significant QTL for either acceptance or survival on pea plants, regional heritability analysis suggested two blocks on chromosome A1 associated with acceptance of pea (50–80 cM and 120–140 cM), in close proximity or overlapping with the regions associated with acceptance of alfalfa (Figure 2). Regional heritability analysis detected no regions associated with survival on either alfalfa or pea plants. All regions identified using this method are shown on Figure 2 and Table 1 and plots showing regional heritability (Vr) estimates across the genome are in Figure S3. We refer to these regions as ‘regional heritability blocks’ (RHBs), to distinguish them from loci identified by r/QTL.

### 3.5 Placement of Chemosensory Genes on Genomic and Linkage Map and Their Association With QTLs

We located 179 functional chemosensory genes (70 ORs, 60 GRs, 19 IRs, 11 OBPs, 10 CSPs and 9 SNMPs) on the four chromosomes. Of these, 60 belonged to regions associated with acceptance phenotypes (Figure 2, see Table S4 for complete list of chemosensory genes with both genomic coordinates and linkage map positions). This is a significant over‐representation of chemosensory genes within QTLs/RHBs (*regioneR* permutation test (Gel et al. 2016): 10000 permutations, observed number of CSGs within acceptance QTLs = 60, expected number = 43.0, *z*‐score = 2.98, SD = 5.69, *p* = 0.002). We had no expectation that survival would be related to chemosensory genes, and no chemosensory genes were located under QTLs/RHBs for survival except where they overlapped with acceptance QTLs. There were 15 chemosensory genes within the putative rearrangement identified on chromosome A1. No CSGs fell within the putative rearrangement on A2.

Members of all categories of chemosensory genes were found under regions associated with acceptance phenotypes: 5 CSPs, 5 GRs, 9 IRs, 1 OBP, 39 ORs and 1 SNMP, but some categories were clearly more strongly represented than others. Of the 60 chemosensory genes falling under regions associated with acceptance phenotypes, 80% were odorant receptors (39 ORs and 9 IRs). This includes 56% of all ORs placed on the linkage map and 47% of IRs; a significant over‐representation of odorant receptors on regions associated with acceptance (Fisher's exact test *p* = 6.429e‐07).

We compared chemosensory genes identified as outliers in four previous studies (Eyres et al. 2016, 2017; Nouhaud et al. 2018; Smadja et al. 2012) with the 179 chemosensory genes mapping to our linkage map. Of these, 81 (45%) have not previously been identified as an outlier, 67 have been identified in a single analysis, 25 in two analyses, four in three analyses and two in all four analyses described here (Table S4). Overall, 24 of the 60 chemosensory genes located within regions associated with plant acceptance have been identified as outliers between host races in at least one previous study (Table 1); compared to the 55% of all CSGs that have been identified as outliers in previous studies, this is not an enrichment. Again, we see an overrepresentation of olfactory genes (16 of the 24, Fisher's exact test *p* = 0.0039). Of the five most repeatedly divergent CSGs (Gr45, Or3 and Or18 in three previous studies, and Or20 and Or21 in all four), four (Gr45, Or3, Or20 and Or21) locate to the start of chromosome A1, within the putative rearrangement and within one regional heritability block for acceptance of pea, and three of these (Or3, Or20 and Or21) also lie within an overlapping regional heritability block for acceptance of alfalfa (Figure 2). The fifth, Or18, is also on chromosome A1, but lies outside the rearrangement and between the regional heritability blocks for acceptance.

### 3.6 Detoxification and Salivary Effector Genes

Six detoxification or salivary effector genes are located under the survival QTL on chromosome A3: cytochrome P450 49a1 (ACYPI003070) (detoxification gene), Gst‐Martin (ACYPI002127) (detoxification gene), S1 (ACYPI000002) (salivary effector), *mrpL16* (ACYPI005001) (salivary effector), *AcypiCht5* (ACYPI009964) (salivary effector) and *Ckiibeta* (ACYPI000089) (salivary effector).

## 4 Discussion

In this study, we have examined the genetic basis of a behavioural phenotype, host acceptance, that acts as a barrier trait and so contributes to reproductive isolation between pea aphid host races. By generating an F2 cross between two host races specialised on *Medicago sativa* and *Pisum sativum* , assaying acceptance behaviour on both host plants, and conducting QTL and regional heritability analyses, we have linked regions of the genome to plant acceptance behaviour and survival phenotypes. We generated an updated linkage map for pea aphids with 790 map positions and have placed 179 chemosensory genes on the four pea aphid chromosomes. By placing chemosensory genes onto our high‐density linkage map, we were able to determine whether specific chemosensory genes relate to the acceptance QTLs and RHBs we identified. Finally, by combining these mapping results with earlier genome scan findings, we were able to confirm a subset of chemosensory genes (with an over‐representation of odorant receptors) as likely candidates for a role in generating behavioural differences between diverging pea aphid host races.

We found a QTL for acceptance of alfalfa in the middle of chromosome A3, which accounts for 15% of the variation in this trait; regional heritability analysis suggested the same region on A3, along with two regions on chromosome A1 (60–90 cM and 100–120 cM) and another olfactory‐receptor‐rich region at the end of the X chromosome. Using regional heritability analysis, we were also able to detect two blocks on chromosome A1 potentially associated with acceptance of pea (50–80 cM and 120–140 cM), in close proximity or overlapping with the regions associated with acceptance of alfalfa. All regions detected had wide confidence intervals, reflecting the size of our F2 family and the heritability of the behavioural traits, which was low (< 0.3) as expected for behavioural traits that typically have large environmental components of variance (Dochtermann et al. 2019). Nevertheless, our analysis has interesting implications.

All regions associated with acceptance of pea co‐localise or lie directly adjacent to regions associated with acceptance of alfalfa; one region associated with acceptance of alfalfa and one for acceptance of pea overlap at the start of chromosome A1, and a second pair of pea and alfalfa acceptance regions are directly neighbouring, further along the same chromosome. Although we only had power to detect the larger‐effect QTLs/RHBs associated with acceptance behaviours, this overlap between regions associated with response to the two host plants suggests that the same regions are involved in host‐plant selection possibly involving fitness trade‐offs at individual chemosensory loci. A similar pattern was observed by Hawthorne and Via (2001) in a cross between North American clones from the alfalfa and clover ( *Trifolium pratense* ) host races. They found only one region associated with acceptance of clover, but it corresponded closely to one of the four QTL for acceptance of alfalfa they identified.

Our analysis had good power to detect QTL of large effect, but much lower power for loci explaining 10% of the variation or less. For behaviour on alfalfa, since we identified no large effect QTL and only a few QTL of medium effect (explaining 7–15% of heritable variation), we can conclude that host acceptance differences must be controlled by at least several genes. Additional QTL of smaller effect were beyond our detection limit unless clustered and so revealed by the regional heritability analysis. For behaviour on pea, we had lower power due to the smaller difference between the parental clones, and we identified no large or medium effect QTL, only RHBs relating to acceptance on pea, suggesting that small effect loci may also underpin aphid response to pea plants. Furthermore, QTL and, especially, genomic regions detected using the regional heritability method may harbour multiple loci, each of small effect, as expected by theoretical work (Yeaman and Whitlock 2011). This is particularly true where chemosensory genes are involved because they are known to be clustered in the genome (Robertson et al. 2019; Sánchez‐Gracia et al. 2009). Our findings are consistent with Hawthorne and Via (2001), who found four acceptance QTL, each of which may contain more than one relevant gene, and who had a similar family size (and so power) to our experiment. In both experiments, the effect sizes are likely to have been over‐estimated due to the Beavis effect (Xu 2003). However, a subsequent analysis by Caillaud and Via (2012) suggested that the number of loci was not large, and a similar conclusion has been reached for host races in several other insects (e.g., *Nilaparvata*, Sezer and Butlin (1998); *Cryptomyzus*, Guldemond (1990); *Rhagoletis*, Dambroski et al. (2005); and see review in Matsubayashi et al. (2010)). Their findings are broadly consistent with theory (e.g., Fry (2003); Matsubayashi et al. (2010)) which suggests that a small number of loci of large effect are more likely to underpin establishment of a population on a new host than a highly polygenic architecture. Our results depart from this theoretical expectation and require some mechanism to maintain linkage disequilibrium among the multiple loci of small effect influencing acceptance (a form of within‐trait coupling, cf. Dopman et al. 2024). This is likely to be facilitated in pea aphid and other insect host races by the spatial separation of habitats, as well as potentially the clustering of loci within the genome and/or positioning within genomic rearrangements.

It is notable that one of the regions associated with acceptance phenotypes was within a large putative complex rearrangement (composed of two inverted and one translocated regions) at the start of chromosome A1. Such rearrangements may trap multiple QTLs of small effect together in linkage disequilibrium or generate tight physical linkage between previously distant loci and so contribute to the maintenance of local adaptation (Berdan et al. 2023; Kirkpatrick and Barton 2006; Yeaman 2013). Further work is needed to establish whether this rearrangement differs in frequency between the alfalfa and pea host races and the origins of this potentially adaptive structural variation (Gompert et al. 2025).

With 790 markers, our new pea aphid linkage map improves upon previous maps. Hawthorne and Via (2001) cited 173 markers across the four linkage groups, and Jaquiéry et al. (2014) mapped 305. The other benefit of this update is the ability to link the markers directly to the genome, enabling us to place 179 chemosensory genes on our linkage map.

Of the 179 chemosensory genes placed on our linkage map, 15 were located within the putative rearrangement and 60 were located within QTLs or RHBs connected to acceptance behaviours, a highly significant enrichment. We looked at the locations of chemosensory proteins, olfactory binding proteins, sensory neuron membrane proteins, ionotropic receptors, olfactory receptors and gustatory receptors, and although members of all categories were represented in regions associated with acceptance phenotypes, there was a marked over‐representation of odorant receptors (39 ORs and 9 IRs, compared to an expectation of 24.2 and 6.6 genes, respectively, if the CSGs were selected at random). While previous work (Caillaud and Via 2000; Schwarzkopf et al. 2013) has shown the importance for the pea aphid to taste the plant to discriminate among potential hosts, plant volatiles or surface factors, and therefore smell, could be particularly important in the decision to spend time probing, as also suggested in the pea aphid (Schwarzkopf et al. 2013) and *Aphis fabae* (Webster et al. 2008). Here, this seems to dominate over the decision to continue feeding, which is more likely to depend on gustatory receptors (Schwarzkopf et al. 2013).

The continuum of divergence among different pea aphid host races offers the possibility to look at the progression of barriers to gene flow through the genome, at varying levels of divergence between races (Peccoud et al. 2014). Fundamental to this is our ability to identify loci responsible for divergent host‐choice decisions between host‐adapted populations, independently from genome scans for barrier effects (Ravinet et al. 2017). The results of previous outlier scans, which identified significantly divergent loci between host race pairs, were prone to false positives and to identifying loci close to the actual targets of selection rather than the direct targets of selection themselves. Genomic regions of the pea aphid genome associated with actual behavioural phenotypes now complement these outlier analyses, narrowing a large number of loci down to a strong set of candidates for involvement in host‐plant choice differences in pea aphids, and in the resulting barriers to gene flow. Many of the genes in acceptance associated regions (24) have been identified in previous outlier scans and expression studies (Eyres et al. 2016, 2017; Nouhaud et al. 2018; Smadja et al. 2012). All 15 chemosensory genes within the large rearrangement identified at the start of chromosome A1 have been identified as outliers in at least one previous study, and four of those (Gr45, Or3, Or20 and Or21) have been identified as outliers on at least three previous occasions. This result suggests a potential role for the rearrangement in maintaining associations between host‐plant preference alleles. Future work needs to establish whether the alternative forms of this rearrangement differ in frequency between the host races. Within the region on chromosome A3 found associated with acceptance of alfalfa by both r/QTL and RH methods, some chemosensory genes were also previously identified with genome scans (Or28, Ir75d.2 and Ir75d.1 on A3). With multiple lines of evidence connecting these 24 genes with divergence between host races and plant acceptance behaviours—genome scans, gene expression studies and now association mapping, this subset of genes provides a particularly robust set of candidates for controlling plant acceptance. Further progress with the functional characterisation of these loci would benefit from genome editing, which is now possible in aphids (Le Trionnaire et al. 2019). However, sequence or expression divergence of chemosensory genes might only be part of the proximal basis of host‐plant acceptance behaviour among pea aphid host races. Although we focused in this study on chemosensory genes that are well identified actors of peripheral chemoreception and well annotated in the pea aphid genome, divergence in signal processing and neuronal pathways might also contribute to this behavioural shift, as shown for olfactory preferences in other insect species (e.g., *Ostrinia nubilalis* : Unbehend et al. 2021; *Rhagoletis pomonella* : Tait et al. 2021; Tait, Batra et al. 2016).

We detected no QTL for survival on pea, which was expected as most *A. pisum* host races perform well on pea initially, but tend not to reproduce on it (Ferrari et al. 2008); furthermore, survival during the first 25 h will not account for mortality due to starvation. More interestingly, we found two QTLs for survival on alfalfa (which is a more selective host for the *A. pisum* host races; Peccoud et al. 2009a): one at the end of chromosome A1, which explained 10% of phenotypic variation, and a second in the middle of chromosome A3, which explained 7% of phenotypic variation. There were no chemosensory genes located under regions associated with survival outside of those also associated with plant acceptance. However, the single QTL for acceptance of alfalfa and one of the two QTLs for survival on alfalfa mapped to overlapping regions of chromosome A3 (Figure 2), implying direct physical linkage between the two traits on alfalfa. Interestingly, this region contains candidate chemosensory genes but also some genes potentially involved in physiological adaptation to host plants, due to their putative functions in detoxification (cytochrome P450, glutathione S transferases) or as salivary effectors (Boulain et al. 2019, 2018; Lu et al. 2016; Simon et al. 2015; Vertacnik and Linnen 2017). This result mirrors the major result of Hawthorne and Via (2001) who found a highly significant co‐localisation of acceptance and fecundity QTLs.

Hawthorne and Via (2001) argued that either functional connections between preference and performance traits via pleiotropy or close linkage between separate loci influencing the two traits would facilitate the maintenance of associations between these traits that is essential for host race formation and possible further evolution of host races towards the completion of speciation. However, outside this study, there is little genetic evidence of such pleiotropy, and other factors may explain the covariance between preference and performance loci (Hardy et al. 2020; Matsubayashi et al. 2010). Phytophagous insects provide an interesting case for understanding how multiple barriers to gene flow can become coupled as host‐choice, host‐associated performance and mating are functionally connected (Forbes et al. 2017; Smadja and Butlin 2011). This linkage between host‐choice and survival loci suggests a further step towards the coupling of multiple barriers to gene flow in the pea aphid genome. We predict that pea aphid host races, and host races more generally, with stronger overall barriers to gene flow will show more evidence for pleiotropy, closely‐linked QTL and chromosomal rearrangements that contribute to tight associations between preference, performance and mating traits.

## Author Contributions

R.B., C.M.S. and J.F. designed the study. J.P. and J.C.S. collected and reared the F0 aphids and generated F1 clones. J.F. and H.F. conducted the F1 cross and performed phenotyping assays. I.E. extracted DNA and generated the RAD sequencing data. I.E., R.B., C.M.S. and J.F. designed and performed the analyses. I.E., R.B., J.F. and C.M.S. wrote the article. All authors commented on draft versions of the manuscript.

## Conflicts of Interest

The authors declare no conflicts of interest.

## Supporting information


Data S1.

## Data Availability

Raw sequence data are available on the EBI short‐read archive (SRA) under bioproject PRJNA1237058. Markers and code used for linkage mapping and linkage map files with genomic coordinates have been archived on Zenodo (10.5281/zenodo.14809563) (Eyres et al. 2025).
