# Supplementary material for: The Genetics of Host Plant Acceptance in Pea Aphids
Source: Mol Ecol. 2025 May 15;34(21):e17795. doi: 10.1111/mec.17795 (PMC12573732; doi:10.1111/mec.17795)
Supplement: Supplementary file 1 — Data S1. [file MEC-34-e17795-s001.zip › PeaAphidLinkageMapSupplementaryFile_April2025.docx]

**Supplementary figures and tables**

**Supplementary table 1.** Summary of sequencing data for F2 mapping cross.

| **ID** | **Total reads** | **Total mapped** | **% mapped** | **Properly paired** | **% properly paired** |
| --- | --- | --- | --- | --- | --- |
| Ms5 | 5,021,662 | 4,202,146 | 83.68 | 3,919,296 | 78.57 |
| Ps3 | 1,458,524 | 1,358,830 | 93.16 | 1,267,600 | 87.54 |
| Ps19 | 3,576,035 | 3,530,536 | 98.73 | 3,279,612 | 92.47 |
| Ms10 | 7,961,960 | 6,806,064 | 85.48 | 6,398,130 | 80.87 |
| Ms5Ps3 | 635,887 | 574,045 | 90.27 | 532,344 | 84.32 |
| Ps19Ms10 | 2,072,756 | 1,525,518 | 73.60 | 1,418,760 | 68.83 |
| Average from ALL | 3,405,048 | 3,268,756 | 95.31 | 3,024,241 | 88.90 |

**Supplementary table 2.** Linkage map statistics.

| Chromosome | **A1** | **A2** | **A3** | **X** | **Total** |
| --- | --- | --- | --- | --- | --- |
| Li *et al* genome | **contig 20849/ NC_042494.1/ CM016664.1** | **contig 21967/ NC_042495.1/ CM016666.1** | **contig 21646/ NC_042496.1/**  **CM016667.1** | **contig 21773/ NC_042493.1/ CM016665.1** |  |
| **Sex/autosome** | A | A | A | X |  |
| **Length (cM)** | 172.044 | 111.873 | 113.964 | 203.859 | 601.74 |
| **Li *et al* chromosome length (Mb)** | 171 | 120 | 42 | 133 | 466 |
| **Number of markers** | 2181 | 2213 | 592 | 1457 | 6443 |
| **Number of positions in map** | 234 | 168 | 129 | 259 | 790 |
| **Markers/pos (mean, median, mode)** | 9.453  4  1 | 13.173  5  1 | 4.589  3  1 | 5.726  3  1 | 10.786  4  1 |
| **Distance between map positions in cM**  **(mean, median, mode)** | 0.7352308  0.521  0.521 | 0.666  0.521  0.521 | 0.8834419  0.521  0.521 | 0.7871004  0.521  0.521 | 0.764  0.521  0.521 |
| **Map positions/Mb** | 1.36 | 1.4 | 3.07 | 1.95 | 1.70 |
| **Recombination rate variation** | Rearrangement at start (0-86 cM)  1.01 cM/Mb across whole length | Suppressed recombination at the start  0.93 cM/ Mb across whole length | 2.17 cM/ Mb across whole length | 1.53 cM/ Mb across whole length | 1.29 cM/ Mb |

**Supplementary table 3.** Significant QTL identified using r/qtl.

| **Phenotype** | **Highest LOD per LG** | **5% sig threshold** | **Significant QTL** | **1.5 LOD support interval** | **% var explained** |
| --- | --- | --- | --- | --- | --- |
| **Acceptance** (counts probing/ counts alive) on **alfalfa** | chr pos lod  c3.loc68 A3 68 7.00 | 3.52 | Chr A3  Pos 68  LOD 7.00  p-val 0.00 | 52.000- 77.766 cM | 15.17% |
| **Survival** (mean duration alive) on **alfalfa – QTL 1** | chr pos lod.p.mu lod.p lod.mu  c1.loc170 A1 171.0 5.03 1.800 4.39 | lod.p.mu = 3.27, lod.p = 2.52, lod.mu = 2.31 | Chr A1  Pos 171  LOD.mu 4.39  p-val 0 | 170.047-172.044 cM | 9.80% for QTL on A1 |
| **Survival** (mean duration alive) on **alfalfa – QTL 2** | chr pos lod.p.mu lod.p lod.mu  marker6037 A3 77.2 3.87 1.576 3.10 | lod.p.mu = 3.27, lod.p = 2.52, lod.mu = 2.31 | Chr A3  Pos 77.2  LOD.mu 3.10  p-val 0 | 66.280-77.766 | 7.02% for QTL on A3 |

**Supplementary figure 1.** boxplots showing acceptance (a and b) or survival (c and d) of aphids from different generations of F2 cross (red = F0_MS grandparental *medicago* biotype; green = F0_PS grandparental *pisum* biotype; teal = F1; purple = F2) when placed on alfalfa (a and c) or pea (b and d) plants.

**Supplementary figure 2.** LOD plots for acceptance of alfalfa (a) and survival on alfalfa (b).

**Supplementary figure 3.** Plots of regional heritability results (Vr) across the genome. Dotted lines show 100 permutations 95% threshold, and red dots indicate blocks where Vr exceded this threshold.
